# Propagation Effect of a Virus Outbreak on a Network with Limited Anti-Virus Ability

**DOI:** 10.1371/journal.pone.0164415

**Published:** 2016-10-27

**Authors:** Yonghong Xu, Jianguo Ren

**Affiliations:** 1 The Key Laboratory of Biotechnology for Medicinal Plants of Jiangsu Province, Jiangsu Normal University, Xuzhou, 221116, China; 2 College of Computer, Jiangsu Normal University, Xuzhou, 221116, China; Shanxi University, CHINA

## Abstract

This paper describes a new computer virus spreading model which takes into account the possibility of a virus outbreak on a network with limited anti-virus ability. Then, the model is investigated for the existence of equilibria and their stabilities are proved and illustrated. Moreover, it is found that these two factors are not only relative to the threshold value determining whether the virus becomes extinct or not, but that they are also relative to the virus epidemic levels. Theoretical and experimental results indicate that, in some ways, it would be practically possible to eradicate the virus or suppress its prevalence below a suitable level. Consequently, some suggestions are proposed that may help eradicate or suppress virus propagation over a real computer network.

## 1. Introduction

Computer viruses are programs that attempt to parasitize themselves on a host and spread to other computers, mainly through the Internet. They have become a great threat to both computer users and network resources. Consequently, the development of appropriate models to understand the mechanisms governing the spread of computer viruses is becoming of importance. Currently, a lot of computer virus propagation models such as *SIR* model [1–3], *SLB* model [4–5], *SEIQV* model [6] as well as their extensions [7–17], have been proposed. These models have mainly been built upon classical epidemic models [18–28] and investigated the behavior of computer virus spread over computer networks by the analogy between computer and biological viruses [29].

To the best of our knowledge, all of the known computer virus models [1–17] that describe virus propagation neglect the fact that viruses possess a paroxysmal nature in common, as demonstrated when a large number of computer viruses have the possibility of an outbreak absence of aura. Furthermore, antivirus techniques always lag behind virus techniques, and thus during that investable lag from the outbreak of a new virus to the widespread application of the anti-virus software aiming to conquer this virus the computer is susceptible to the attack. As a result, the virus would not be entirely eradicated, but instead be temporarily suppressed and therefore still capable of infecting other computers, which may lead to huge losses. Almost all of the existing models assume, first, that during the time [*dt*, *t+dt*], once infected, any susceptible computer is in its latency (i.e., all of the *S* computers become the *E* ones), and secondly, that by using the anti-virus software on the affected network, the virus can be completely and immediately eliminated. However, these assumptions are inconsistent to the facts that, in general, most viruses break out suddenly. Zuo et al [30] have shown that there is no any perfect anti-virus software that can detect and clear all kinds of viruses.

This paper pays attention to the spreading behaviors of a computer virus during a virus outbreak on a network with limited anti-virus ability. Accordingly, a new computer virus propagation model is proposed, which incorporates those two kinds of new state transitions. First, we give the threshold value *R*_0_, which determines whether the virus disappears completely or not, and find that the dynamic behavior of the proposed model is determined by it. Next, the proposed model admits a virus-free equilibrium which is globally asymptotically stable that results in virus eradication if *R*_0_≤1 and admits a virus equilibrium which is globally asymptotically stable whereas *R*_0_>1. System parameters are considered to analyze the dynamical behaviors of the model. It is found that (1) the outbreak probability and transmission rate due to the limited anti-virus ability are strongly relative to the threshold value *R*_0_, and (2) the outbreak probability and transmission rate are heavily connected with the virus epidemic levels. These results imply that, to some degree, it would be practically possible to eradicate the virus or inhibit its prevalence below a suitable level. Finally, theoretical and experimental studies reveal the effect of system parameters on virus propagation. Consequently, some suggestions are made that may help suppress virus propagation over a computer network.

In the next section, we establish a mathematical model to be discussed. In Section 3, we study the stability of the virus-free and virus equilibrium of the model, respectively. The given parameters are considered to analyze the dynamic behaviors of the model. In Section 4, we use theoretical and experimental research to reveal the effect of parameters on virus propagation to which corresponding suggestions are made. Finally, some conclusions are given in Section 5.

## 2. Mathematical Model

Based on the computer virus propagation models involving the *S*, *E*, *I* and other compartments in Ref. [31–33] and ignoring infection details, the total number of computers connected the Internet are divided into four compartments:

1. Susceptible compartments (*S*):The set of all external uninfected computers that are connected to the network, i.e. susceptible computers.
2. Exposed compartments (*E*): The set of all latent computers, i.e. infected computers where all viruses are latent.
3. Infected compartments (*I*): The set of all infected computers where all of the viruses are currently breaking out.
4. Recovered compartments (*R*): The set of all recovered computers that have run the anti-virus software.

Let *S*(*t*), *E*(*t*), *I*(*t*) and *R*(*t*) denote their numbers at time *t*, respectively. The involved parameters *b* denotes the rate at which external computers are connected to the network, *β* denotes the rate at which, when having a connection to one latent computer, one susceptible computer can become latent one, *γ* denotes the rate at which one latent computer breaks out, *α* denotes the recovery rate of infected computers and depends on the ability of the anti-virus software, *μ* denotes the rate at which one computer is removed from the network. Furthermore, since most viruses break out suddenly, with the time period [*dt*, *t+dt*], due to a possible connection with infected computers, every susceptible computer is either latent with probability *(1-p) βI(t)dt*, or breaks out with probability *pβI(t)dt*, where *p*>0 is a constant. Since the existing anti-virus ability of any given network is limited, the virus is temporarily suppressed with probability *ε*, where *ε* > 0 is a constant. At time *t*, every infected computer is either latent sequentially with probability *ε* or recovered with probability *α*.

From the work above, the transmission between the model can be expressed by the following system of differential equations:

$$\left\{ \begin{array}{l} \frac{dS}{dt}=b-\beta SI-\mu S,\\ \frac{dE}{dt}=(1-p)\beta SI-\gamma E+\varepsilon I-\mu E,\\ \frac{dI}{dt}=p\beta SI+\gamma E-\varepsilon I-\alpha I-\mu I,\\ \frac{dR}{dt}=\alpha I-\mu R. \end{array}\right.$$ (1)

Because the first three equations in (1) are independent of *R*, we can consider the following reduced model:

$$\left\{ \begin{array}{l} \frac{dS}{dt}=b-\beta SI-\mu S,\\ \frac{dE}{dt}=(1-p)\beta SI-\gamma E+\varepsilon I-\mu E,\\ \frac{dI}{dt}=p\beta SI+\gamma E-\varepsilon I-\alpha I-\mu I. \end{array}\right.$$ (2)

With initial conditions *S*(0) ≥ 0, *E*(0) ≥ 0, *I*(0) ≥ 0 and all the parameters are positive constants. It is easily verified that the set

$$\Omega =\{(S,E,I):S\geq 0,E\geq 0,I\geq 0,S+E+I\leq \frac{b}{\mu }\}$$ (3)

is positively invariant for this system.

## 3. Model Analysis

This section is devoted to understanding the dynamic behaviors of the model (2). First, we will obtain a threshold value *R*_*0*_ for the model and it is defined as the number of virus-free computers that are infected by a single infected computer. It is easy to see that the model (2) always admits a virus-free equilibrium *E*_0_ = (*S*_0_, 0, 0). Then let

$$\varpi =\left[\begin{array}{c} (1-p)\beta SI\\ p\beta SI \end{array}\right],\nu =\left[\begin{array}{l} \;(\gamma +\mu )E-\varepsilon I\\ (\varepsilon +\mu +\alpha )I-\gamma E \end{array}\right].$$ (4)

Then,

$$\begin{array}{l} F=\text{Jacobian of}\;\varpi \;\text{at}\;E_{0}=\left[\begin{array}{l} 0\;(1-p)\beta S_{0}\\ 0\;p\beta S_{0} \end{array}\right].\\ V=\text{Jacobian of}\;\nu \;\text{at}\;E_{0}=\left[\begin{array}{l} \gamma +\mu \;-\varepsilon \\ \;-\gamma \;\varepsilon +\mu +\alpha \end{array}\right]. \end{array}$$

Hence, next generation matrix for the model is

$$K=FV^{-1}=\left[\begin{array}{l} \frac{\gamma (1-p)\beta S_{0}}{(\mu +\gamma )(\mu +\varepsilon +\alpha )-\varepsilon \gamma }\;\frac{(\mu +\gamma )(1-p)\beta S_{0}}{(\mu +\gamma )(\mu +\varepsilon +\alpha )-\varepsilon \gamma }\\ \frac{\gamma p\beta S_{0}}{(\mu +\gamma )(\mu +\varepsilon +\alpha )-\varepsilon \gamma }\;\frac{(\mu +\gamma )p\beta S_{0}}{(\mu +\gamma )(\mu +\varepsilon +\alpha )-\varepsilon \gamma } \end{array}\right].$$ (5)

Again, the spectral radius *R*_0_ of the matrix *K* is the basic reproduction number of the model, i.e.

$$R_{0}=\rho (K)=\frac{\gamma (1-p)\beta S_{0}+(\mu +\gamma )p\beta S_{0}}{(\mu +\gamma )(\mu +\varepsilon +\alpha )-\gamma \varepsilon }=\frac{\beta S_{0}(\gamma +\mu p)}{(\mu +\gamma )(\mu +\varepsilon +\alpha )-\gamma \varepsilon }.$$ (6)

### 3.1 The virus-free equilibrium and its stability

Model (2) always has a unique equilibrium $E_{0}=(\frac{b}{\mu },0,\;0)$. Then the characteristic equation of the linearization of model (2) near *E*_0_ is

$$\det(\begin{array}{l} -\mu -\lambda \;0\;-\beta S_{0}\\ 0\;-\gamma -\mu -\lambda \;(1-p)\beta S_{0}+\varepsilon \\ 0\;\gamma \;p\beta S_{0}-\varepsilon -\alpha -\mu -\lambda \end{array})=0.$$ (7)

Clearly, the eigenvalues are −*μ* and other roots are decided by the following equations:

$$f(\lambda )=\lambda^{2}+a\lambda -a_{1},$$ (8)

where

$$\begin{array}{l} a=\mu +\gamma -p\beta S_{0}+\varepsilon +\alpha +\mu ,\\ a_{1}=(\mu +\gamma )(p\beta S_{0}-\varepsilon -\alpha -\mu )+\gamma (1-p)\beta S_{0}+\varepsilon \gamma . \end{array}$$ (9)

If *R*_0_ < 1, then *a* = *μ* + *γ* − *pβS*_0_ + *ε* + *α* + *μ* > 0, and

$$\begin{array}{l} a_{1}=(\mu +\gamma )(p\beta S_{0}-\varepsilon -\alpha -\mu )+\gamma (1-p)\beta S_{0}+\varepsilon \gamma \\ \;=[(\mu +\gamma )(\varepsilon +\alpha +\mu )-\gamma \mu ](R_{0}-1)<0. \end{array}$$ (10)

The two roots of Eq (9) both have negative real parts, by the Hurwitz criterion we can obtain the following result:

#### Theorem 3.1

When *R*_0_ < 1, the virus-free equilibrium *E*_0_ is local stability.

Now, it is turn to examine the global stability of virus-free equilibrium by use of the Lyapunov direct method. The following Theorem is obtained:

#### Theorem 3.2

When *R*_0_ ≤ 1, the virus-free equilibrium *E*_0_ is global stability.

**Proof**. Define

V(E,I)=γE+(μ+γ)I (11)

then

$$\begin{array}{l} \dot{V}(E,I)=\gamma (1-p)\beta SI+\gamma \varepsilon I+(\gamma +\mu )p\beta SI-(\gamma +\mu )(\varepsilon +\alpha +\mu )I\\ \;=I[\beta S(\gamma +\mu p)-(\gamma +\mu )(\varepsilon +\alpha +\mu )+\gamma \varepsilon ]\\ \;\leq I[\beta S_{0}(\gamma +\mu p)-(\gamma +\mu )(\varepsilon +\alpha +\mu )+\gamma \varepsilon ] \end{array}$$ (12)

As all the model parameters are positive, it follows that $\dot{V}(E,I)<0$ for *R*_0_ < 1 with $\dot{V}(E,I)=0$ if and only if *I* = 0 or *R*_0_ = 1. Hence, *V* is a lyapunov function on Ω. Thus, *I* → 0 as *t* → ∞. *I* = 0 in the first equation of model (2) shows that *S* → *b* / *μ* as *t* → ∞. Therefore, it follows from the Lasalle’s invariance principle that every solution of the model, under the initial conditions in Ω, approaches *E*_0_ as *t* → ∞.

**Fig 1:**
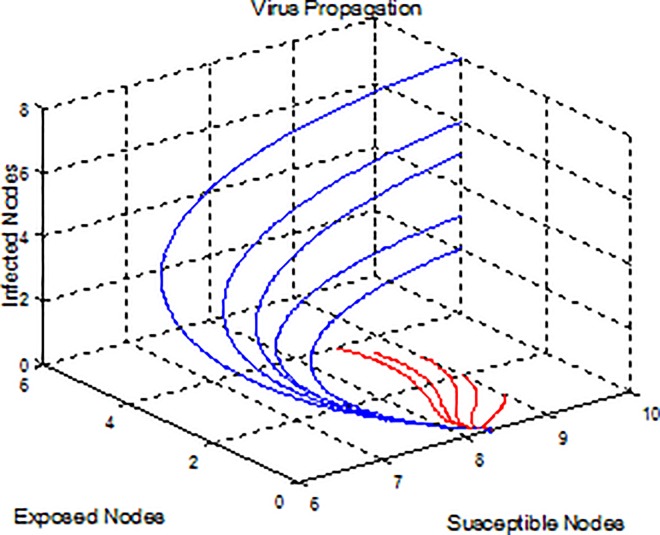
A plot indicates that the viruses can become extinct.

**Example 1.** Consider model (2) with parameters b = 5, β = 0.07, μ = 0.6, γ = 0.25, ε = 0.3, p = 0.7 and R_0_≈0.57<1. The condition of virus-free equilibrium stability is satisfied and hence the virus-free equilibrium *E*_0_(8.33,0,0) is asymptotically stable (see Fig 1), i.e. the virus infection can become extinct after some period of time.

### 3.2 The virus equilibrium and its stability

Now, let us investigate the existence and stability of virus equilibrium of model (2). By calculations, the unique equilibrium E* is given by

$$\begin{array}{l} S^{*}=\frac{(\alpha +\mu )(\gamma +\mu )+\varepsilon \mu }{\beta (\mu p+\gamma )},\\ E^{*}=\frac{(\varepsilon +\alpha +\mu )I^{*}-p\beta S^{*}I^{*}}{\gamma },\\ I^{*}=\frac{b\beta (\mu p+\gamma )-\mu (\alpha +\mu )(\gamma +\mu )+\varepsilon \mu^{2}}{(\alpha +\mu )(\gamma +\mu )+\varepsilon \mu }, \end{array}$$ (13)

And the corresponding characteristic equation of the linearization of model (2) is

$$\det(\begin{array}{l} -\beta I^{*}-\mu -\lambda \;0\;-\beta S^{*}\\ \;(1-p)\beta I^{*}\;-\gamma -\mu -\lambda \;(1-p)\beta S^{*}+\varepsilon \\ \;p\beta I^{*}\;\gamma \;p\beta S^{*}-\varepsilon -\mu -\alpha -\lambda \end{array})=0,$$ (14)

which equals the following equation:

$$f(\lambda )=\lambda^{3}+a_{1}\lambda^{2}+a_{2}\lambda +a_{3},$$ (15)

where

$$a_{1}=(\beta I^{*}+\mu )(\gamma +\mu )-(p\beta S^{*}-\varepsilon -\alpha -\mu )>0,$$ (16)

$$a_{2}=(\beta I^{*}+\mu )(\mu +\gamma )-(\beta I^{*}+\mu )(\mu +\gamma )(p\beta S^{*}-\varepsilon -\alpha -\mu )+p\beta S^{*}\beta I^{*}+p\gamma \beta S^{*}-\gamma \beta S^{*}-\varepsilon \gamma >0,$$ (17)

$$a_{3}=\gamma (1-p)\beta S^{*}\beta I^{*}+\beta S^{*}(\gamma +\mu )p\beta I^{*}-(\beta I^{*}+\mu )(\gamma +\mu )(p\beta S^{*}-\varepsilon -\alpha -\mu )-\gamma (\beta I^{*}+\mu )[(1-p)\beta S^{*}+\varepsilon ]>0.$$ (18)

It is easy to verify that *a*_1_*a*_2_–*a*_3_ > 0 holds, then according to the Routh-Hurwitz criterion, the following theorem is given:

#### Theorem 3.3

Virus equilibrium E* is locally asymptotically stable when *R*_0_ > 1.

**Remark 1.** This Theorem expresses that, under the assumption of virus outbreak on a network with limited anti-virus ability, virus epidemic would attain a certain level, and the model admits no the periodic and chaotic dynamic behaviors.

#### Theorem 3.4

Virus equilibrium E* is globally asymptotically stable.

**Proof**. We consider the following Lyapunov function constructed as

$$V(t)=[\frac{(\alpha +\mu )(\gamma +\mu )+\varepsilon \mu }{\beta S^{*}}]\int_{S^{*}}^{S}\frac{t-S^{*}}{t}dt+\gamma \int_{E^{*}}^{E}\frac{t-E^{*}}{t}dt+(\gamma +\mu )\int_{I^{*}}^{I}\frac{t-I^{*}}{t}dt.$$ (19)

Meanwhile, for simplicity, denote

$x=\frac{S}{S^{*}},y=\frac{E}{E^{*}},z=\frac{I}{I^{*}},$ then time derivative of *V*(*t*) along the solution of Eq (2) is

$$\begin{array}{l} V(t)'=[\frac{(\alpha +\mu )(\gamma +\mu )+\varepsilon \mu }{\beta S^{*}}](\frac{S-S^{*}}{S})[b-\beta SI-\mu S]\\ \;+\gamma (\frac{E-E^{*}}{E})[(1-p)\beta SI+\varepsilon I-(\gamma +\mu )E]\\ \;+(\gamma +\mu )(\frac{I-I^{*}}{I})[p\beta SI+\gamma E-(\varepsilon +\alpha +\mu )I]\\ \;=[\frac{(\alpha +\mu )(\gamma +\mu )+\varepsilon \mu }{\beta S^{*}}][b-\beta S^{*}I^{*}xz-\mu S^{*}x-b\frac{1}{x}+\beta S^{*}I^{*}z+\mu S^{*}]\\ \;+\gamma [(1-p)\beta S^{*}I^{*}xz+\varepsilon I^{*}z-(\gamma +\mu )E^{*}y-(1-p)\beta \frac{1}{y}xzS^{*}I^{*}-\varepsilon \frac{1}{y}zI^{*}+(\gamma +\mu )E^{*}]\\ \;+(\gamma +\mu )[p\beta S^{*}I^{*}xz+\gamma E^{*}y-(\varepsilon +\alpha +\mu )I^{*}z-p\beta S^{*}I^{*}x-\gamma E^{*}\frac{1}{z}y+(\varepsilon +\alpha +\mu )I^{*}] \end{array}$$ (20)

By using the following equations

$$\left\{ \begin{array}{l} b-\beta S^{*}I^{*}-\mu S^{*}=0,\\ (1-p)\beta S^{*}I^{*}-\gamma E^{*}+\varepsilon I^{*}-\mu E^{*}=0,\\ p\beta S^{*}I^{*}+\gamma E^{*}-\varepsilon I^{*}-\alpha I^{*}-\mu I^{*}=0, \end{array}\right.$$ (21)

Eq (20) can be written as

$$\begin{array}{l} V(t)'=[\frac{(\alpha +\mu )(\gamma +\mu )\mu +\varepsilon \mu^{2}}{\beta }+(\gamma +\mu )p\beta S^{*}I^{*}](2-x-\frac{1}{x})+\gamma \varepsilon I^{*}(2-\frac{z}{y}-\frac{y}{z})\\ \;+\gamma (1-p)\beta S^{*}I^{*}(3-\frac{1}{x}-\frac{xz}{y}-\frac{y}{z}) \end{array}$$ (22)

Since the arithmetical mean is great than or equal to the geometrical mean, thus, $2-x-\frac{1}{x}\leq 0$ for *x* > 0 and the equality holds if and only if *x* = 1; $2-\frac{z}{y}-\frac{y}{z}\leq 0$ for *y*,*z* > 0 and the equality holds if and only if *y* = *z*; $3-\frac{1}{x}-\frac{xz}{y}-\frac{y}{z}\leq 0$ for *x*,*y*,*z* > 0 and the equality holds if and only if *x* = *y* = *z*. Therefore, *V*(*t*)' ≤ 0 for *x*,*y*,*z* > 0 and *V*(*t*)' = 0 holds if and only if *x* = 1 and *y* = *z*. Thus, it follows from LaSalle Invariance Principle that the virus equilibrium E* is globally asymptotically stable.

**Fig 2:**
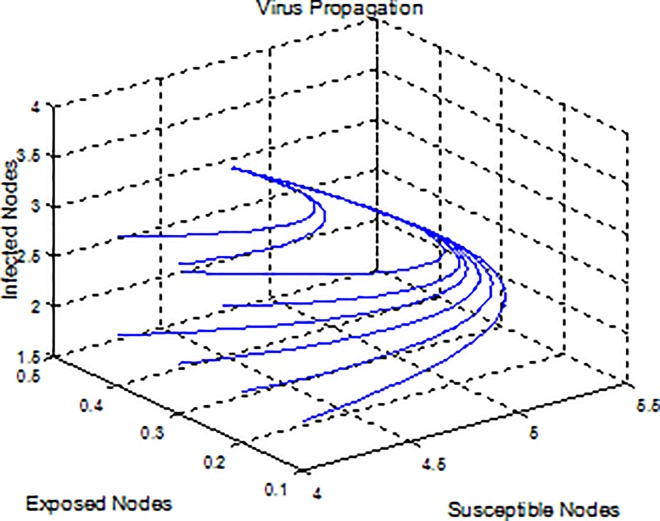
A plot indicates that the viruses become steady.

**Example 2.** Consider model (2) with parameters b = 5, β = 0.15, μ = 0.6, ε = 0.03, p = 0.9, γ = 0.4 where *R*_*0*_ = 1.90>1 satisfies the stable conditions for the virus equilibrium (4.38, 0.34, 3.60), and hence the virus equilibrium is asymptotically stable (see Fig 2), i.e., the virus can maintain its infection to some extent.

**Fig 3:**
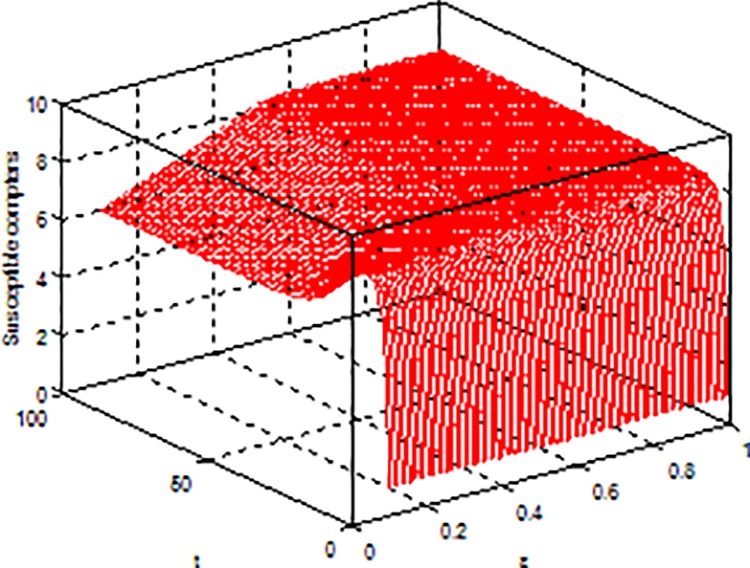
Dynamical behaviors of every system variable with parameters*ε-S(t*.*)*.

**Fig 4:**
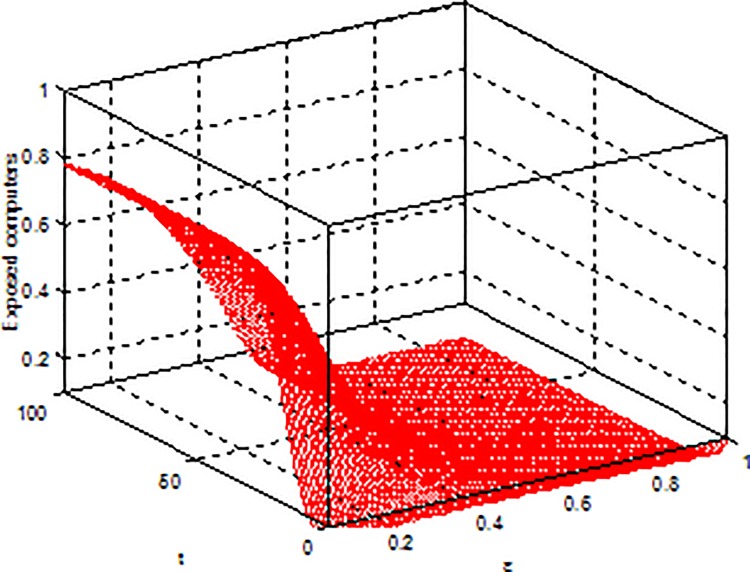
Dynamical behaviors of every system variable with parameters*ε-E(t)*.

**Fig 5:**
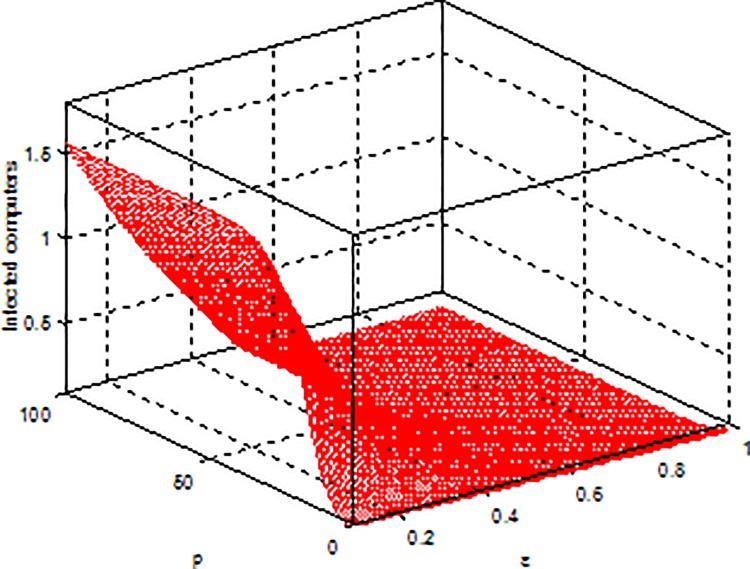
Dynamical behaviors of every system variable with parameters*ε-I(t)*.

**Example 3.** Consider model (2) with parameters b = 5, β = 0.15, μ = 0.6, p = 0.5, γ = 0.5, the global dynamical behavior of every variables of the model (2) with varying parameter*ε*is shown in Fig 3*ε-S(t)*, Fig 4*ε-E(t)* and Fig 5*ε-I(t)*.

**Fig 6:**
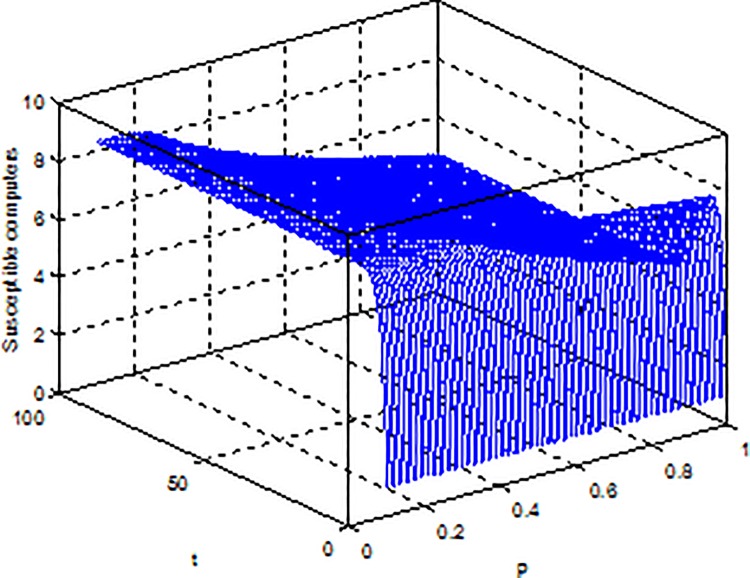
Dynamical behaviors of every system variable with parameters *p-S(t)*.

**Fig 7:**
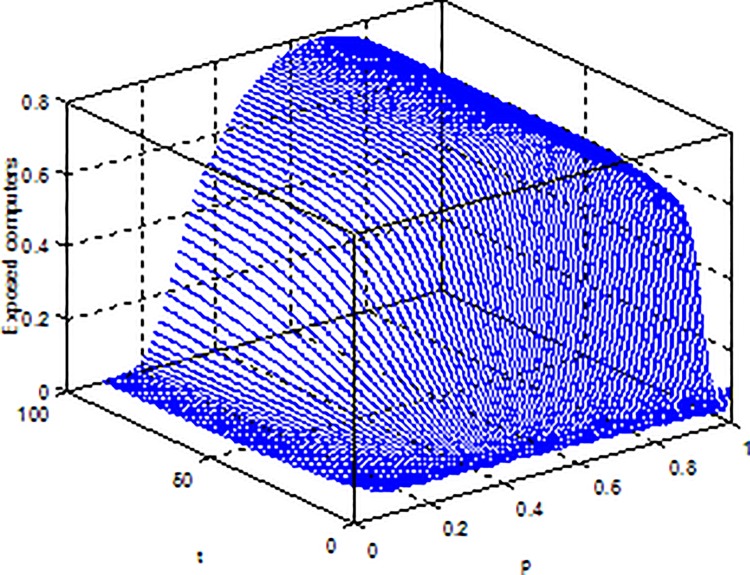
Dynamical behaviors of every system variable with parameters *p-E(t)*.

**Fig 8:**
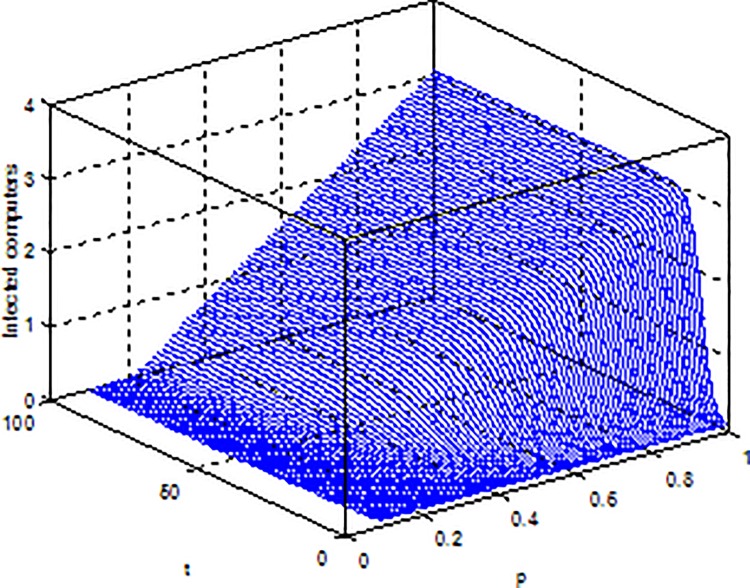
Dynamical behaviors of every system variable with parameters *p-I(t)*.

**Example 4.** Consider model (2) with parameters b = 5, β = 0.15, μ = 0.6, ε = 0.2, γ = 0.5, the global dynamical behavior of every variables of the model (2) with varying parameter *p* is shown in Fig 6 *p-S(t)*, Fig 7 *p-E(t)* and Fig 8 *p-I(t)*.

## 4. Further Discussions and Suggestions

Theorem 3.2 and 3.3 tell us, to some degree, it would be possible to eradicate the virus or inhibit its epidemics below a proper level in practice. In order to do this, it is critical to have an overall knowledge of the effects of parameters on the virus propagation. By the proof of Theorem 3.1, we have

$$R_{0}=\rho (K)=\frac{\beta S_{0}(\gamma +\mu p)}{(\mu +\gamma )(\varepsilon +\alpha +\mu )-\gamma \varepsilon }=\frac{\beta S_{0}(\gamma +\mu p)}{(\mu +\gamma )(\alpha +\mu )+\varepsilon \mu }.$$ (23)

Since breakout probability *p* and transmission rate*ε* play a key role, then differentiating *R*_0_ with respect to them, we can obtain:

$$\frac{\partial R_{0}}{\partial p}=\frac{\mu \beta S_{0}}{(\mu +\gamma )(\alpha +\mu )+\varepsilon \mu },\;\frac{\partial R_{0}}{\partial \varepsilon }=\frac{-\mu \beta S_{0}(\gamma +\mu p)}{{\left[(\mu +\gamma )(\alpha +\mu )+\varepsilon \mu \right]}^{2}}<0.$$ (24)

Hence, we obtain the following Theorem:

**Theorem 4.1** Consider model (2), the following claims hold true

1. *R*_0_ is increasing with *p*, and is decreasing with *ε*.

**Fig 9:**
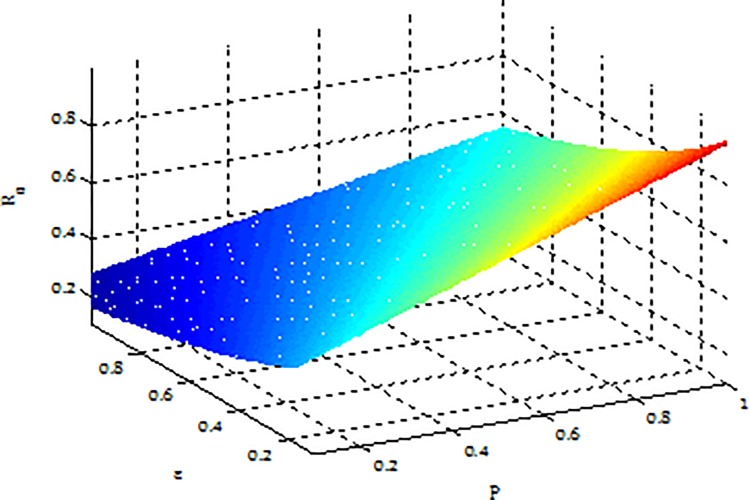
An illustration of effect of *p* and*ε*on *R*_*0*_.

**Example 5.** Consider the effect of*ε*, *p* on *R*_0_ in model (2) with parameters b = 5, β = 0.075, μ = 0.6, γ = 0.5, Fig 9 shows that *R*_0_ is increasing with *p* and decreasing with*ε*.

Next, we are ready to study the effect of parameters on virus epidemic level I*. A direct calculation yields:

$$\frac{\partial I^{*}}{\partial p}=\frac{b\beta \mu }{(\alpha +\mu )(\gamma +\mu )+\varepsilon \mu }>0,\frac{\partial I^{*}}{\partial \varepsilon }=\frac{-\mu b\beta (\mu p+\gamma )}{{\left[(\alpha +\mu )(\gamma +\mu )+\varepsilon \mu \right]}^{2}}<0.$$ (25)

**Theorem 4.2** Consider model (2), the following claims hold true

1. I* is increasing with *p* and decreasing with *ε*.

**Fig 10:**
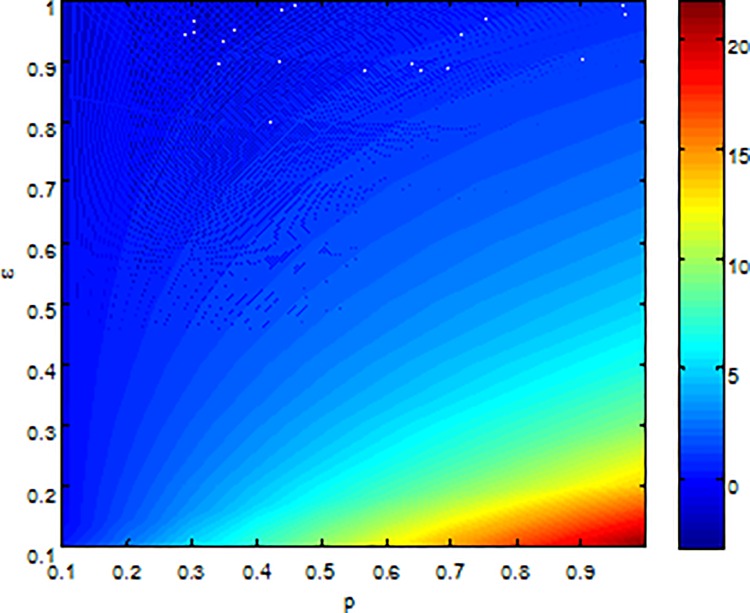
An illustration of effect of *p* and*ε*on I*.

**Example 6.** Consider the effect of*ε*, *p* on I* in model (2) with parameters b = 5, β = 0.15, μ = 0.6, γ = 0.5, Fig 10 shows that I* is increasing with *p* and decreasing with *ε*, respectively.

Based on analyses above, the following suggestions may help eliminate or suppress the spread of computer viruses in a real network:

1. When *R*_0_ is well below one, Theorem 4.2 demonstrates that *R*_*0*_ is more sensitive to change in *p* than in*ε*. This sensitivity informs us that outbreak is more important than passive detection for computer viruses, which leads us to eliminate the virus by dissecting its logical structure [34], analysis of function of every module and their interrelation so as to uncover the source of the virus burst.
2. When *R*_0_ is significantly greater than one, it leads us to suppress virus propagation to an acceptable level (i.e. decreasing the number of infected computers I* by either updating the anti-virus timely or reinstalling the system to dispel the bias of anti-virus software).

## 5. Conclusions

By taking into consideration the possibility of a virus outbreak on a network with limited anti-virus ability, a new computer virus spreading model is proposed and analyzed. The results are as follows:

1. The outbreak probability and transmission rate are strongly relative to the threshold value *R*_0_, which determines whether the virus may become extinct or not. More specifically, *R*_0_ is proportional to the former and inversely proportional to the latter.
2. The outbreak probability and transmission rate are heavily connected with the virus epidemic level I*. More specifically, I* increases with the former and decreases with the latter.epidemic levels
3. The virus-free equilibrium is globally stable if *R*_0_ ≤ 1 whereas the virus equilibrium is globally stable if *R*_0_ > 1.

These results indicate that, in some ways, it would be practically possible to eradicate the virus or suppress its prevalence below an acceptable level. Theoretical and experimental results reveal the effects of the parameters on virus propagation. Consequently, the proposed suggestions may help eradicate or suppress virus propagation over a real computer network.

The main goal of this paper is to better understand the propagation of computer virus qualitatively rather qualitatively. In our view, our work give an insight into the investigation of computer virus spreading by incorporating the objective realistic factors into compartment modeling, which could attract growing attention and interest. Meanwhile, the proposed model is based on the homogeneous network, which can be expanded into heterogeneously node-based model in complex networks, and it is one of our next objectives.
